# Feasibility of Tomotherapy-Based Image-Guided Radiotherapy to Reduce Aspiration Risk in Patients with Non-Laryngeal and Non-Pharyngeal Head and Neck Cancer

**DOI:** 10.1371/journal.pone.0056290

**Published:** 2013-03-07

**Authors:** Nam P. Nguyen, Lexie Smith-Raymond, Vincent Vinh-Hung, Paul Vos, Rick Davis, Anand Desai, Thomas Sroka, Dave Abraham, Shane P. Krafft, Michelle Stevie, Homayoun Modarresifar, Beng-Hoey Jo, Misty Ceizyk

**Affiliations:** 1 Department of Radiation Oncology, University of Arizona, Tucson, Arizona, United States of America; 2 Department of Radiation Oncology, University Hospitals of Geneva, Geneva, Switzerland; 3 Biostatistics, East Carolina University, Greenville, North Carolina, United States of America; 4 Department of Radiation Oncology, University of Texas at M. D. Anderson, Houston, Texas, United States of America; 5 Department of Radiation Oncology, Darmouth College, Hanover, New Hampshire, United States of America; 6 Department of Radiology University of Arizona, Tucson, Arizona, United States of America; The University of Chicago, United States of America

## Abstract

**Purpose:**

The study aims to assess the feasibility of Tomotherapy-based image-guided radiotherapy (IGRT) to reduce the aspiration risk in patients with non-laryngeal and non-hypopharyngeal cancer. A retrospective review of 48 patients undergoing radiation for non-laryngeal and non-hypopharyngeal head and neck cancers was conducted. All patients had a modified barium swallow (MBS) prior to treatment, which was repeated one month following radiotherapy. Mean middle and inferior pharyngeal dose was recorded and correlated with the MBS results to determine aspiration risk.

**Results:**

Mean pharyngeal dose was 23.2 Gy for the whole group. Two patients (4.2%) developed trace aspiration following radiotherapy which resolved with swallowing therapy. At a median follow-up of 19 months (1–48 months), all patients were able to resume normal oral feeding without aspiration.

**Conclusion and Clinical Relevance:**

IGRT may reduce the aspiration risk by decreasing the mean pharyngeal dose in the presence of large cervical lymph nodes. Further prospective studies with IGRT should be performed in patients with non-laryngeal and non-hypopharyngeal head and neck cancers to verify this hypothesis.

## Introduction

Dysphagia and aspiration are common following radiotherapy for head and neck cancer [1]. Aspiration is potentially life-threatening as the cough reflex may be absent or ineffective following head and neck cancer radiotherapy. Head and neck cancer survivors with chronic aspiration following treatment may develop anxiety and depression because of social isolation, which may severely impact on their quality of life. Recent studies suggest a correlation between radiation dose to the pharyngeal constrictor muscles and the risk of tube feeding dependence following head and neck radiotherapy [2], [3]. New radiotherapy techniques such as intensity-modulated radiotherapy (IMRT) may decrease dysphagia severity and the need for gastrostomy tubes because of decreased radiation dose to critical structures for swallowing. Some institutions advocate shielding the larynx separately with a midline block in the split-field (SF) IMRT technique to reduce radiation dose to the larynx and the middle and inferior constrictor muscles [4]. Since aspiration risk increases with pharyngeal dose, the prevalence of aspiration should be reduced following head and neck cancer treatment in those that have a decreased dose to this region. However, in the presence of cervical lymph nodes, a mid-line laryngeal block can under-dose the cervical lymph nodes in close proximity to the larynx, leading to regional recurrences [5]. Whole-field (WF) IMRT is often advocated in the presence of cervical lymph nodes to ensure adequate target coverage [6]. However, studies have demonstrated WF-IMRT to deliver higher laryngeal and pharyngeal radiation dose, leading to unnecessary complications such as laryngeal edema and aspiration. In a previous dosimetric study comparing WF-IMRT to Tomotherapy-based image-guided radiotherapy (IGRT), we had demonstrated that IGRT may significantly reduce the larynx, middle and inferior pharyngeal doses in non-laryngeal and non-hypopharyngeal cancer without compromising target coverage [7]. The dosimetric advantage of IGRT to spare laryngeal edema correlated with a significant reduction in the rate of severe laryngeal edema and improvement in the quality of the voice in patients treated with IGRT [8]. The current retrospective study was performed to determine if low radiation doses to these swallowing structures could decrease the aspiration rate in this subset of head and neck cancer patients as well.

## Materials and Methods

The medical records of 48 patients undergoing helical Tomotherapy-based IGRT for head and neck cancer in non-laryngeal and non-hypopharyngeal sites at the University of Arizona Department of Radiation Oncology were retrospectively reviewed following institutional review board (IRB) approval. The University of Arizona IRB waived the patient consent requirement because of the retrospective nature of this study, which was limited to patient chart reviews. Laryngeal and hypopharyngeal cancers were excluded from the study because they are associated with a high rate of aspiration at diagnosis [9]. Prior to treatment, each patient was simulated in a supine position with a head and neck Aquaplast mask for treatment immobilization. A computed tomography (CT) scan with and without intravenous (IV) contrast for treatment planning was performed in the treatment position. The head and neck areas from the vertex to the mid thorax were outlined with a slice thickness of 3 mm. CT scan with IV contrast was employed to enhance target volume delineation. Radiotherapy planning was performed on the CT scan without contrast to avoid possible interference of contrast density on radiotherapy isodose distributions. Diagnostic positron emitting tomography (PET)-CT scan imaging was also incorporated with the CT planning when available. A 0.5 cm bolus was placed on any area of the skin involved by the tumor and on any palpable cervical lymph nodes. Normal organs at risk for complications were outlined for treatment planning, including the spinal cord, brain stem, cochlea, mandible, parotid glands, larynx, pharyngeal muscles, eyes, and oral cavity. The tumor and grossly enlarged lymph nodes on CT scan (CTV1) with a margin (PTV1) were treated to 70 Gy in 35 fractions (2 Gy/fraction). The margins were 0.5–1 cm circumferentially around CTV1 depending on the anatomic location. The areas at high risk (PTV2) were defined as at least 1 cm around the gross tumor and pathologic cervical lymph nodes. The areas at low risk (PTV3) were defined as the subclinical regional lymph nodes with 0.5 cm margins circumferentially. PTV2 and PTV 3 were treated to 63 Gy and 56 Gy in 35 fractions respectively. Patients undergoing postoperative radiation were treated to 66 Gy, 59.4 Gy, and 54 Gy in 33 fractions to PTV1, PTV2, and PTV3, respectively. Minimal target coverage was 95% for all targets with at least 99% of the prescribed dose delivered to 100% of PTV1. The lymph nodes in the ipsilateral neck, including the retropharyngeal lymph nodes, were treated to the base of skull if there was any cervical lymph node enlargement or PET – positive lymph nodes. Contralateral uninvolved lymph nodes were treated prophylactically with the C1 vertebral body as the superior border of the radiation field.

In the case of bilateral cervical lymph node involvement, both necks were treated to the base of skull to avoid any marginal misses. Mean dose to the parotid was kept below 2600 cGy if there was no ipsilateral cervical lymph node enlargement. Dose constraints for other normal organs at risk (OAR) were: spinal cord (45 Gy), brain stem (50 Gy), optic chiasm (45 Gy), mandible (70 Gy to less than 30% of the mandible). Doses to larynx and pharyngeal muscles for non-laryngeal and non-hypopharyngeal cancers were kept between 20–40 Gy if feasible, as it is our strict policy that all PTV targets should be covered by at least the 95% target dose. The larynx and pharyngeal muscles were contoured from the hyoid bone (superior border) to the cricoid cartilage (inferior border) following consultation with a radiologist. The pharyngeal muscles outlined included the middle and inferior pharyngeal constrictors muscles according to the guidelines developed by Eisbruch et al [10].

The larynx and pharyngeal muscles would have been effectively shielded from radiation with a laryngeal block in the conventional supraclavicular field of the SF -IMRT technique [4].

It is our recommendation that all head and neck cancer patients in our institution have a modified barium swallow (MBS) immediately prior to radiotherapy to exclude silent aspiration. The MBS was performed in the Department of Speech Pathology by speech pathologists who were blinded to the patient's cancer stage. However, they were instructed that these patients were at risk of aspiration. Patients with demonstrated aspiration on MBS who were scheduled to undergo concurrent chemoradiation had percutaneous gastrostomy (PEG) tube placement in anticipation of severe weight loss from mucositis. Repeat MBS was performed four to six weeks after treatment completion to assess aspiration risk. Although some patients continued to experience mucositis at four weeks following chemoradiation, excessive fibrosis may decrease the effectiveness of swallowing therapy if MBS was delayed [11]. Swallowing therapy and PEG tube feedings were recommended for patients who developed aspiration after radiotherapy. Patients with severe weight loss during treatment also continued with PEG tube feedings. All patients had PET-CT scans four and ten months after completion of treatment in addition to regularly scheduled follow-up consisting of physical exam and nasopharyngoscopy if indicated. The PEG tube was removed if there was no evidence of aspiration on post-treatment MBS, no evidence of disease on PET-CT scan after treatment, and if the patient had recovered from any treatment-related weight loss.

Patients with loco-regional recurrence underwent surgical salvage, and the PEG tube was not removed in anticipation of severe dysphagia post -surgery. All patients were monitored by a team of experienced dietitians to assess their nutritional needsfollowing treatment. The presence or absence of dysphagia, continued weight loss, and other parameters such as total protein, albumin, and pre-albumin were taken into consideration before the decision to remove the PEG tube even in the absence of aspiration was made.

## Results

Among 170 head and neck cancer patients treated at the University of Arizona Department of Radiation Oncology from 2007 to 2012, we identified 48 patients with non-laryngeal and non-hypopharyngeal cancers who had both MBS immediately prior to and following radiotherapy. The patients selected did not have aspiration observed on the MBS prior to radiotherapy, although they may have been experiencing abnormal swallowing because of the cancer and/or previous surgery. Median age at diagnosis was 57 years (range: 25–83 years). Forty-four were males and four were females. The disease site distribution was: oropharynx (24), oral cavity (12), parotid (4), unknown (2), nasopharynx (3), maxillary sinus (2) and neck recurrence (1). The two patients that had an unknown primary also had submental lymph node metastases, which were resected. In both cases, the primary was presumed to be an occult oral cavity lesion and the larynx was not included in the radiotherapy fields. The three patients that had a parotid primary also had cervical lymph node metastases requiring bilateral neck irradiation. One patient had locally advanced maxillary sinus cancer which invaded into the cheek and also required bilateral neck irradiation. Another patient had locally advanced maxillary sinus cancer and gross lymph nodes metastases in the left neck. Breakdown by stage demonstrated one stage I, six stage II, 13 stage III, 16 stage IVA, ten stage IVB, one stage IVC, and one patient with recurrence.

Definitive concurrent chemoradiation was delivered to twenty-eight patients. Thirteen patients received postoperative chemoradiation, four patients had postoperative radiotherapy alone, and three patients had definitive radiotherapy alone.

Indications for postoperative chemoradiation were positive or close margins after surgery or extracapsular lymph node extension. Table 1 summarizes the patient characteristics.

**Table 1:** Patient characteristics.

Patient Number		48
Age	Median	57
	Range	25–83
Sex	Male	44
	Female	4
Squamous Histology		48
Tumor Sites	Oropharynx	24
	Oral cavity	12
	Parotid	4
	Unknown (submental metastases)	2
	Nasopharynx	3
	Paranasal sinus (maxillary)	2
	Neck recurrence	1
Stages	I	1
	II	6
	III	13
	IVA	16
	IVB	10
	IVC	1
	Recurrence	1
T stages	Tx	2
	T1	7
	T2	13
	T3	10
	T4	15
	Recurrence	1
Neck nodes	N0	13
	N1	15
	N2	14
	N3	6
Treatment	Postoperative radiation	4
	Radiotherapy alone	3
	Postoperative chemoradiation	13
	Chemoradiation	28
Follow-up (months)	Median	19
	Range	1–48

The mean pharyngeal dose was 23.2 Gy (15.4–54 Gy) for the whole group. The most common abnormalities observed on pre-treatment MBS were decreased transit in the oral phase (5 patients), pooling in the vallecula (9 patients) and decreased laryngeal elevation in the pharyngeal phase of swallowing (3 patients). Following radiotherapy, two patients (4.6%) developed trace aspiration which resolved with swallowing therapy. Mean pharyngeal dose was 17.8 Gy and 19.3 Gy for the two patients with aspiration. The most common abnormalities observed on post-treatment MBS were decreased transit or residue in the oral phase (9 patients), decreased laryngeal elevation (4 patients), reduced base of tongue contraction (8 patients), reduced epiglottic inversion (4 patients), and pooling in the vallecula (13 patients). No patient developed aspiration pneumonia following treatment.

At a median follow-up of 19 months (1–48 months), all patients were able to resume normal oral feedings without aspiration. Among the 38 patients who had prophylactic PEG tube placement for chemoradiation (three declined tube placement), three remained dependent on PEG tube feedings because of severe weight loss, two were able to resume oral feedings but their PEG tubes were kept because of the short follow-up (three months), and one patient had severe dysgueusia and continued tube feedings even though he had no dysphagia. The other 32 patients had their PEG tubes removed four to 10 months after treatment. The delay in removing the PEG tube was recommended by the dietitians to ensure that the patients achieved their ideal body weight because of the severe weight loss and/or chronic dysgueusia which prevented the patient from having adequate nutrition. The three patients who declined prophylactic PEG tube placement did not have to undergo tubes feedings during or after treatment.

## Discussion

**Figure 1:**
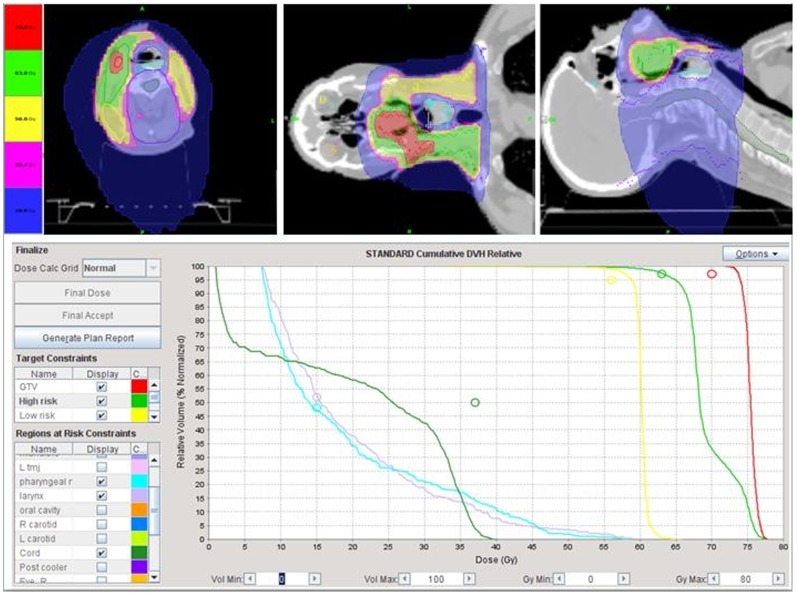
Illustration of the potential of Tomotherapy to spare the pharyngeal muscles in a patient with locally advanced base of tongue cancer and right neck nodal metastases treated with definitive concurrent chemoradiation. Despite the proximity of the gross tumor and neck nodes treated to 70 Gy, mean pharyngeal muscle radiation dose was 22.5 Gy. A split field intensity-modulated radiotherapy technique to shield the larynx and pharyngeal muscles would have underdosed the right neck nodes and gross tumor. The patient is in clinical remission two years following treatment and has no difficulty with swallowing except for xerostomia as the parotid gland could not be spared.

**Figure 2:**
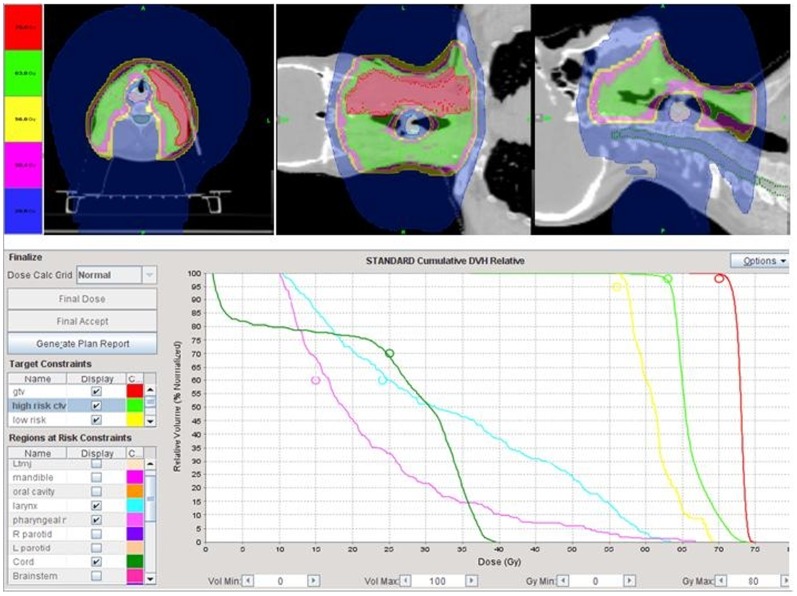
Illustrating the potential of Tomotherapy to spare the pharyngeal muscles in a patient who had postoperative chemoradiation for locally advanced base of tongue and bilateral neck metastases. Even though the right neck was dissected and required radiation of the surgical bed and scars to 63 Gy, the pharyngeal muscles can still be spared from excessive radiation dose. The midline laryngeal block with the split field intensity-modulated radiotherapy technique would have had underdosed the surgical scar and area of the surgical bed located in close proximity to the larynx and the gross lymph nodes on the left side. The patient is in remission 13 month after treatment.

To our knowledge, despite the relatively small number of patients, this is the first study to report aspiration rate following a pharyngeal musculature-sparing IGRT technique. No patient in the study had aspiration at cancer diagnosis. Thus, the prevalence of aspiration following radiotherapy was dependent on radiation dose delivered to the swallowing structures. All patients had WF-IGRT with the goal to limit the pharyngeal dose. We used the WF-IGRT technique in the study to provide adequate dose delivery to the low neck in the presence of cervical node metastases and to reduce the risk of low neck recurrences associated with a split field technique [5]. Mutliple studies have correlated the radiation dose to the pharyngeal muscles to the presence of severe dysphagia and/or aspiration. Fua et al [12] used the Common Toxicity Criteria (CTC) scale to grade the severity of dysphagia following WF-IMRT and SF-IMRT for nasopharyngeal cancers. Patients who had CTC grade 3 required tube feedings. The WF-IMRT was associated with a higher dose to the pharyngeal muscles and severe dysphagia. Severe dysphagia and prolonged tube feedings were reported more often in the WF-IMRT cohort (mean pharyngeal dose 55.2 Gy) compared to the SF-IMRT group (mean pharyngeal dose 27.2 Gy). The corresponding median time to insertion of the feeding tube was 36 days and 38 days, respectively. The mean pharyngeal dose in our study was 23.2 Gy, similar to the SF-IMRT. In another study, Caglar et al [3] investigated the aspiration risk following WF-IMRT of 96 patients with head and neck cancer of all anatomic sites, including laryngeal and hypopharyngeal cancers (18%). MBS was performed four to six weeks following treatment. There was no pretreatment MBS. Thus, the prevalence of silent aspiration pre-treatment could not be assessed in a population of patients which might be at a high risk for aspiration because of the tumor subsites included in the study. Aspiration was assessed according to the Swallowing Performance Scale (minimal aspiration: grade 5, severe aspiration: grade 6–7). Following treatment, 32% of the patients developed grade 5–7 aspiration. It was unclear how many patients developed grade 5 aspiration. However, a high aspiration rate was observed when the radiation dose to the inferior pharyngeal muscles exceeded 52 Gy. High dose to the pharyngeal muscles was associated with WF-IMRT and the inclusion of patients with laryngeal and hypopharyngeal cancers. Thus, the methodology performed by Caglar et al was very similar to our study because the swallowing study was performed four to six weeks after treatment. We do not distinguish between minimal and severe aspiration because our aim was to correlate aspiration rate and radiation dose to the pharyngeal muscles. However, we demonstrated that IGRT by virtue of its rapid dose fall-off compared to WF-IMRT may significantly reduce pharyngeal muscle dose even in the presence of cervical lymph node involvement and can significantly reduce the aspiration risk. Figure 1 and 2 illustrated the ability of IGRT to spare the larynx and pharynx from a high radiation dose, though the involved adjacent cervical lymph nodes were treated to a curative dose of radiation. Nguyen et al [13] recently reported excellent regional control in head and neck cancer patients treated with WF-IGRT in the presence of cervical lymph nodes. Only one out of 76 patients with either unilateral or bilateral cervical lymph nodes developed regional recurrences. In another study correlating MBS before and three months after WF-IMRT for oropharyngeal (31) and nasopharyngeal (5) cancer, aspiration rate was 47% at three months after treatment [14]. Mean dose to the pharyngeal constrictors was 64 Gy. All patients who developed aspiration received more than 60 Gy to the pharyngeal constrictors. Two other studies also corroborated the correlation between high radiation dose to the pharyngeal muscles and dysphagia three months and 20 months after treatment [15], [16]. Table 2 summarizes these studies correlating dysphagia and aspiration with pharyngeal muscle dose.

**Table 2:** Mean pharyngeal dose (Gy) correlation with dysphagia severity or aspiration reported in the literature.

Study	Mean pharyngeal dose (Gray)	Critical Structures implicated	Clinical endpoints	Time-frame following treatment
**Caglar et al ** [**3**]	>52	Inferior constrictors larynx	Aspiration (32%)	4–8 weeks
**Fua et al ** [**12**]	55.2	Middle constrictors Inferior constrictors	Prolonged tube feedings (median time: 38 days)	36–38 days
**Feng et al ** [**14**]	>60	Superior constrictors Supraglottic larynz	Aspiration (47%)	3 months
**Levendag et al ** [**15**]	48–51	Superior constrictors Middle constrictors	QOL questionnaires for dysphagia	3 months
**Dirix et al ** [**16**]	50	Middle constrictors Inferior constrictors Supraglottic larynx	QOL questionnaires for dysphagia	20 months

NA: not assessed; QOL: quality of life.

In studies reporting aspiration rates following either 3D-CRT, SF-IMRT, or WF-IMRT in patients with non-laryngeal and non-hypopharygeal cancers, the rates of aspiration ranged from 6.4–54% [17]–[21]. If we excluded the study of Schwartz et al [20] who used the SF-IMRT technique, aspiration rates ranged from 16–54%. Table 3 summarizes the reported aspiration rates in the literature for non-laryngeal and non-hypopharyngeal cancers.

**Table 3:** Aspiration rate reported in the literature following radiotherapy for non-laryngeal and non-hypopharyngeal head and neck cancer.

Study	Patient Number	Anatomic site	Treatment Modality	Technique	Aspiration rate
**Nguyen et al ** [**17**]	46	oropharynx	chemoradiation	C	54%
**Nguyen et al ** [**18**]	18	oropharynx	postoperative therapy	C	50%
**Langerman et al ** [**19**]	13	oral cavity	chemoradiation	NS	23%
	18	oropharynx			44%
**Feng et al ** [**13**]	36	oropharynx	chemoradiation	IMRT	44%
		oral cavity		WF	
**Schwartz et al ** [**14**]	31	oropharynx	chemoradiation	IMRT	6.4%
				SF	
**Feng et al ** [**15**]	73	oropharynx	chemoradiation	IMRT	16–26%
				WF	

C: conventional with two lateral and a supraclavicular field; NS: not specified; IMRT: intensity-modulated radiotherapy; WF: whole-field; SF: split-field; IGRT: image-guided radiotherapy.

Although controversy still exists about which swallowing structures are critical and need to be spared to prevent long-term dysphagia and aspiration, the studies that reported the least amount of dysphagia and aspiration used techniques which effectively decreased radiation dose to the larynx, and middle and inferior pharyngeal constrictor muscles [12], [20]. Thus, WF-IGRT, by virtue of its laryngeal and pharyngeal muscle sparing along with its decreased aspiration rate may be the technique of choice for non-hypopharyngeal and non-laryngeal head and neck cancer [8]. The additional advantage of WF-IGRT is the excellent regional control in the presence of cervical lymph nodes because of the delivery of high dose to these structures in the face of laryngeal and pharyngeal muscle sparing [13].

We should point out that swallowing is a complex mechanism requiring a perfect coordination of multiple muscles within the tongue, pharynx, and larynx. Functional alteration of any of these structures may result in dysphagia and aspiration. Reduced tongue strength, tongue base retraction, and delayed laryngeal vestibule closure were commonly observed following chemoradiation for head and neck cancer [22]. Thus, shielding of the larynx and pharyngeal muscles may reduce the risk of aspiration but complete elimination of aspiration following radiotherapy may not be achieved because of high radiation dose to other swallowing structures such as the base of tongue. As an illustration, the two patients in our study who developed trace aspiration after radiotherapy both had mean pharyngeal dose less than 20 Gy. However, they did not require PEG tube feeding as their aspiration resolved with swallowing therapy. At a median follow-up of 19 months, all study patients were able to resume normal oral feeding without evidence of aspiration. Long-term PEG tube feeding was frequently linked to dysgueusia, which is commonly observed after chemoradiation for head and neck cancer [23].

The limitations of the present study include the retrospective nature of the study, the heterogeneity of the patient population and treatment modalities, the small number of patients, and the fact that we did not include patients with laryngeal or hypopharyngeal cancer. However, in such patients, it would not be feasible to spare the pharyngeal muscles from excessive radiation because of the close proximity of these tumor subsites, which often demonstrate invasion of the muscles in locally advanced stages. Nevertheless, despite these caveats, sparing of the pharyngeal musculature from excessive radiation may decrease aspiration rate, and merits further investigation. Prospective studies with large numbers of patients should be performed to assess the impact of WF-IGRT on dysphagia and aspiration in patients with head and neck cancer.

## Conclusion

Tomotherapy-based IGRT may reduce the aspiration rate for non-laryngeal and non-hypopharyngeal head and neck cancer patients because of the decreased pharyngeal muscle dose. Prospective studies should be performed to assess the potential of IGRT to reduce treatment dysphagia and to possibly improve patient quality of life.
